# Evaluation of different concentrations of eugenol (*Syzigium aromaticum*) on electrocardiography of nile tilapia (*Oreochromis niloticus*, L. 1758)

**DOI:** 10.1007/s11259-026-11198-0

**Published:** 2026-04-10

**Authors:** Clarissa Araújo da Paz, Axell Lins, Luciana Eiró Quirino, Daniella Bastos de Araujo, Thaysa de Sousa Reis, Gabriela Brito Barbosa, Rayllan da Cunha Ferreira, Yris da Silva Deiga, Luana Vasconcelos de Souza, Marcelo Victor dos Santos Brito, Moisés Hamoy

**Affiliations:** 1 Laboratory of Pharmacology and Toxicology of Natural Products, Biological Sciences Institute, Federal University of Pará (UFPA), Belém, PA Brazil; 2 Federal University of Pará, R. Augusto Corrêa, Guamá, Belém, 01 - 66075-110 Pará Brazil

**Keywords:** *Oreochromis niloticus*, Eugenol, Electrocardiography, Cardiac physiology, Euthanasia, Fish welfare

## Abstract

The present study evaluated the effects of increasing concentrations of eugenol on cardiac electrophysiological activity in *Oreochromis niloticus* (Nile tilapia) to establish a humane and effective euthanasia protocol. Forty-five fish (24.38 ± 2.5 g) were randomly assigned to five groups: control, vehicle (70% ethanol), and three eugenol treatments (700, 800, and 900 µL L⁻¹). Electrocardiographic (ECG) recordings were obtained using silver electrodes and a high-impedance amplifier for 30 min. Cardiac parameters, including heart rate, QRS amplitude, and PQ, RR, and QT intervals, were analyzed using ANOVA and Tukey’s tests. A concentration-dependent decrease in heart rate and ECG amplitude was observed. The 700 µL. L⁻¹ group showed a 53.8% reduction in heart rate compared with the control, whereas 800 µL. L⁻¹ and 900 µL. L⁻¹ induced complete cardiac arrest within 30 min of exposure. These results demonstrate that eugenol exerts a dose-dependent depressive effect on cardiac excitability in *O. niloticus*, supporting its use as a humane euthanasia agent for experimental and aquaculture settings. The findings provide electrophysiological evidence of cardiac inhibition induced by high eugenol concentrations, contributing to the refinement of fish welfare protocols.

## Introduction

Aquaculture has undergone significant technological changes, driven by the need to ensure food security and improve human nutrition (Ahmed and Thompson 2019). In this commercial context, fish have become an important alternative to meet this need, with Nile tilapia (*Oreochromis niloticus*) being one of the fish species with the greatest potential due to the profitability of its production (Yuan et al. 2017). According to the FAO, global tilapia production is expected to reach 7.3 million tons by 2030 (Munguti et al. 2022).

In fish farming, handling and transportation are stressful factors for animals that can have negative impacts on health and growth, which has motivated the search for effective anesthetics to prevent such effects (Fernandes et al. 2017; Santos et al. 2020).

With research using fish as experimental models, the need for appropriate ways to sacrifice the animals at the end of the studies has become evident, either to ensure the animal’s well-being or because the study objectives have been achieved (von Krogh et al. 2021). In this sense, euthanasia has become more studied and is generally carried out by overdose with immersion anesthetics (Neiffer and Stamper 2009).

Eugenol can be obtained from natural plant oils and is the most important component of clove oil (S*yzigium aromaticum*) (Marchese et al., 2017). This compound, by acting biologically in different ways, has a wide range of possible applications and pharmacological uses (Ulanowska & Olas 2021) such as anesthesia in fish (Batiha et al. 2020; Fernandes et al. 2017). In addition, it is a natural product that presents a high degree of safety for use in tilapia (da Paz et al. 2024).

The high efficacy observed in fish is due to its high affinity for GABA receptors, resulting in the blockage of nociception, muscle relaxation, and loss of postural reflexes (Nuanmanee et al. 2024; Meyer and Fish 2008; Guénette et al. 2007; Zeng et al. 2024). After absorption through the gills, the anesthetic quickly passes through tissues, crossing the blood-brain barrier, which inhibits activity at neuronal synaptic membranes, causing muscle relaxation and a decrease in cardiac output (Zahl et al. 2012).

Electrophysiological methods are a powerful electrical phenotyping tool considered indispensable for the electrophysiological characterization of fish in vivo (Zhao et al. 2019), in view of their satisfactory application for studying the mechanism of ion transport through epithelial tissues, by interpreting the measurements as an electrical circuit (Bakker 1985). Currently, many anesthetic agent are used as anesthetics and are said to be effective for one species or another, however, behavioral changes alone are not enough to determine the real physiological state of the animal, such as cardioventilatory responses (Barbas et al., 2021).

This study is the first to evaluate the effects of high concentrations of eugenol using electrophysiological tools to assess cardiac function during the euthanasia procedure in O. niloticus specimens, and to evaluate acute cardiotoxicity and the main morphographic changes in the electrocardiogram during the euthanasia induction process.

## Materials and methods

### Animals

*O. niloticus* (*n* = 45), with an average weight of 24.38 ± 2.5 g, were used. They were kept in aquariums at the Laboratory of Pharmacology and Toxicology of Natural Products at the Federal University of Pará - Belem- Brazil (LFTPN - ICB - UFPA) in a controlled temperature environment (25 to 28 °C) and a 12 h C: 12 h E photoperiod. The animals were fed twice a day with commercial feed (32% protein) until they reached satiety. At the same time, the aquariums were siphoned for cleaning, removing leftover food and animal excrement, as well as partially renewing the water (about 20% of the tank volume every 24 h) with water from the same source. During the fish’s acclimatization period (which will last 20 days), water quality variables such as temperature (°C), hydrogen potential (pH), dissolved oxygen (DO), ammonia (NH_3_^+^) and total hardness was monitored and have been preserved according to the following values: Temp 26.8 °C; pH 7.5; DO > 5.0 mg/L; NH_3_^+^ 0.1 mg/L and total hardness 70 NTU. All procedures were approved by the ethics committee (Ethics Committee on the Use of Animals Federal University of Pará - Institute of Biological Sciences) (CEUA/ UFPA N 3900030624).

### Obtaining the anesthetic agent, preparation and preservation

**Fig. 1:**
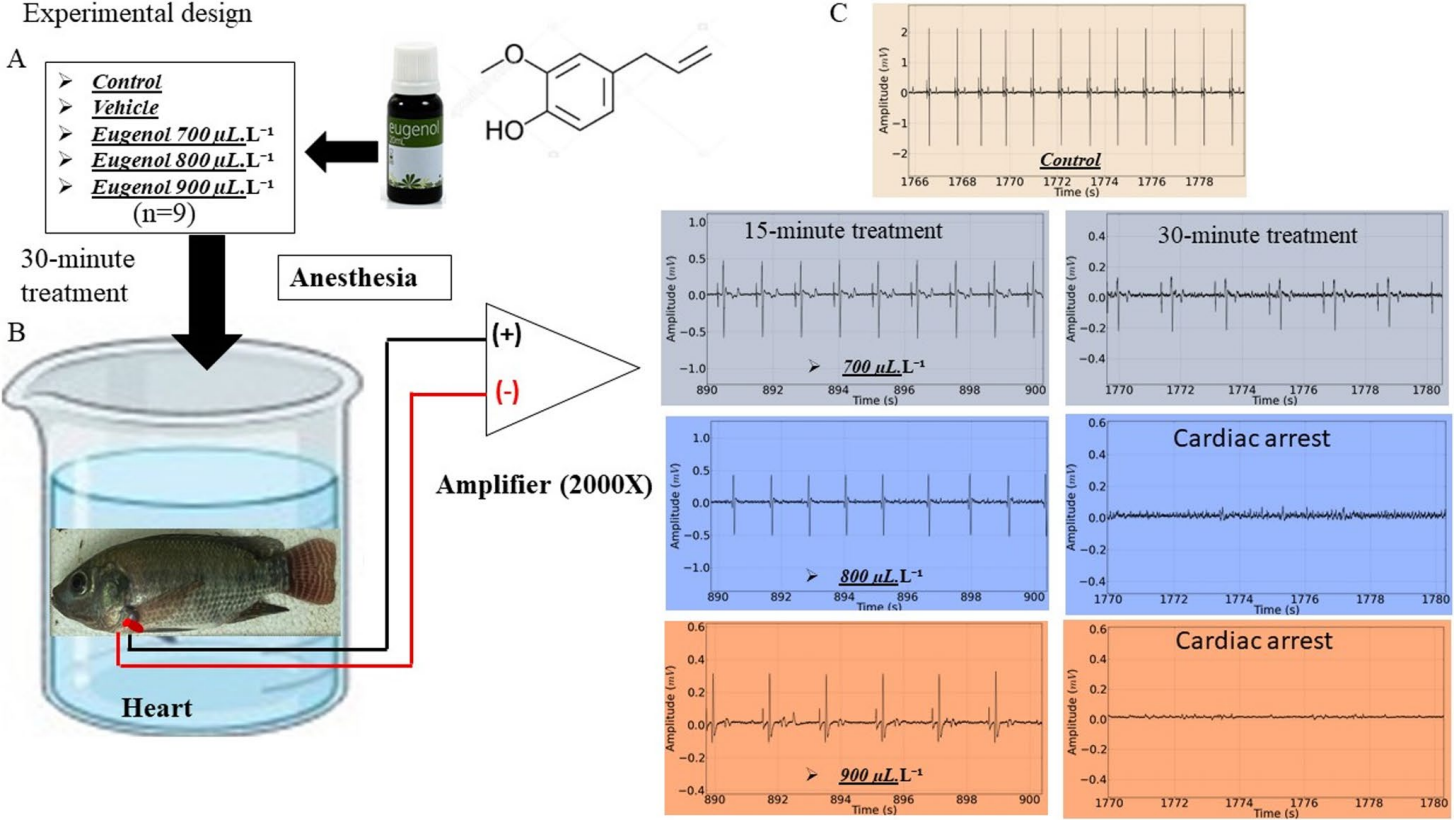
Describes the experimental design with the following phases: Dilution of the anesthetic for the treatment of the animals (**A**); recording lasting 30 min at different concentrations of eugenol (**B**) and recording obtained during each treatment evaluated at the end of 15 and 30 min of treatment with eugenol (**C**)

The eugenol (U.S.P. 99–100%) was obtained from the laboratory of the Biodynamic company (Parque Industrial, Ibiporã, Paraná- Brazil), in 20 ml. The matrix solution was made at a concentration of 10%, being 1 ml of eugenol for 9 ml of 70% alcohol (Ethyl alcohol), later the doses were removed for each experimental group (Fig. 1A). The anesthetic agent, diluted in alcohol, was stored in a 10 ml syringe and later placed in water through a 0.45 × 13 mm needle to promote greater dispersion in the water. The stock solution was stored in a 50 ml Falcon tube and stored under refrigeration at LFTPN-ICB - UFPA.

### Description of the experiment (ECG)

Tilapia specimens were subjected to euthanasia tests with an overdose of anesthetic agent applied for 30 min, during which the electrocardiogram was recorded to assess cardiac activity or cardiac silencing (cardiac arrest recorded) (Fig. 1B). The animals were randomly distributed into the following groups: (a) Control group (*n* = 9), (b) Vehicle group (9 ml of 70% ethyl alcohol) (*n* = 9), (c) Group of animals subjected to eugenol at concentrations of 700 µL. L^− 1^ (*n* = 9), (d) 800 µL. L^− 1^ (*n* = 9) and (e) 900 µL. L^− 1^ (*n* = 9) (Fig. 1A).

### Obtaining an electrocardiogram (ECG)

For the analysis and monitoring of cardiac function, silver 925 electrodes with a diameter and length of 0.3 mm and 5.0 mm, respectively, were made in a non-conjugated manner. For the ECG, the position used to fix the reference electrode, which followed the indication of the cardiac vector, was fixed in the ventral opercular left portion of the tilapia, 0.2 mm before the end of the opercular cavity (da Paz et al. 2024). The recording electrode was inserted 2.0 mm from the right opercular opening, it was possible to capture the cardiac D1 derivation, as shown below in Fig. 1B. The electrodes were then connected to a high-impedance amplifier (Grass Technologies, Model P511) to acquire 30-minute recordings. Each recording was analyzed in two periods: 890–900 s (initial 15 min of recording) and 1770–1780 s (end of 30 min of recording) (Fig. 1C). Once the periods were obtained, the data was analyzed as follows: Heart rate (bpm), amplitude (mv) and duration of the QRS complex (ms) and the duration of the R-R (ms), P-Q (ms) and S-T (ms) intervals. The energy level was then assessed using power intensity spectrograms.

### Recording and analysis of records

The ECG electrodes were connected to a digital data acquisition system using a high-impedance differential input amplifier (Model P511, Grass Technologies, USA), which was configured for 0.3 and 300 Hz filtering and 2000X amplification, and were monitored using an oscilloscope (ProteK, Model 6510). The recordings occurred continuously and were digitized at a rate of 1 kHz on a computer with a data acquisition card (National Instruments, Austin, USA) and were stored on a hard disk for later processing using specialized software (LabVIEW express). To minimize external interference, the entire recording acquisition process was conducted inside a Faraday cage made of metallic material. All analyses are performed up to 40 Hz, as the electrical network has noise in the recordings at 60 Hz. (TMC™) (Barbas et al., 2021; De Souza et al. 2019; Cantanhêde et al. 2021).

Analysis of the recordings obtained was made possible by advising a tool to be built using Python programming version 2.7. For mathematical processing, the Numpy and Scipy libraries was used and, for graphing, the Matplolib library was used. The graphical interface was developed using the PyQt4 library (Souza-Monteiro et al. 2015). The amplitude graphs will show the potential differences between the reference and recording electrodes. In addition, the recording signals were observed at 1000 samples per second.

### Statistical analysis

The normality and homogeneity of the variations were checked using the Kolmogorov-Smirnov and Levene tests, respectively. As the residuals were normally distributed and the variations were equal, comparisons between the mean amplitude of the tracings and the control values were made using ANOVA followed by Tukey. Mean values were followed by their respective standard deviation values (mean ± SD). The minimum level of significance was set at *p < 0.05, **p < 0.01 and ***p < 0.001 in all cases. GraphPad^®^ Prism 5 software was used for statistical tests. 

### Ethical approval and accordance

All procedures involving fish were conducted in accordance with institutional, national, and international ethical guidelines for the use of animals in research, including the Basel Declaration and the ARRIVE guidelines. The experimental protocol was reviewed and approved by the Ethics Committee on the Use of Animals of the Federal University of Pará (CEUA/UFPA, protocol number 3900030624). The study complied with the recommendations for the euthanasia of aquatic species described by the American Veterinary Medical Association (AVMA 2020).

## Results

**Fig. 2:**
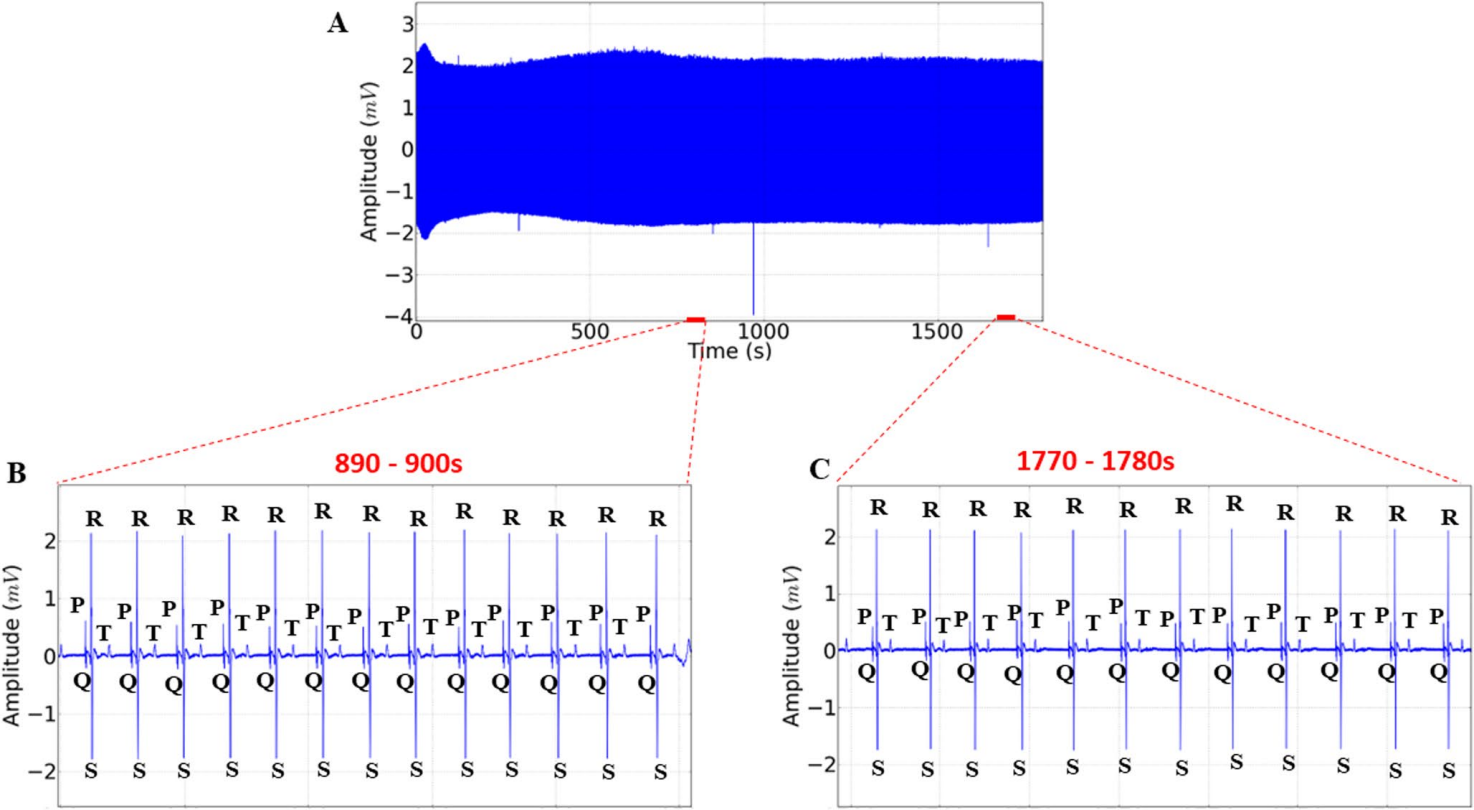
**-** Electrocardiographic recordings showing cardiac activity in juvenile tilapia over a 30-minute period (**A**), amplification of the final 10 s of contact over 15 min (890 to 900s), with indication of the characteristic morphographic elements of the electrocardiogram (**B**), and amplification of the 10-second recording (1770–1780 s) during a 30-minute immersion bath, with indication of the morphographic elements of the electrocardiogram (**C**)

The heart rate in the control group averaged 78.74 ± 4.87 bpm in the first 15 min of recording, with sinus rhythm and the presence of all cardiac stimuli on the electrocardiogram (Fig. 2A and B). The heart rate in the final 30 min for the control group was 78.89 ± 3.88 bpm, with the presence of all characteristic stimuli on the electrocardiogram and maintenance of sinus rhythm (Fig. 2A and C). In the 10-second amplification, all components of the recordings could be observed, such as the P wave, the QRS complex, and the T wave, which made it possible to evaluate the intervals during the immersion bath in groups treated with eugenol.

**Fig. 3:**
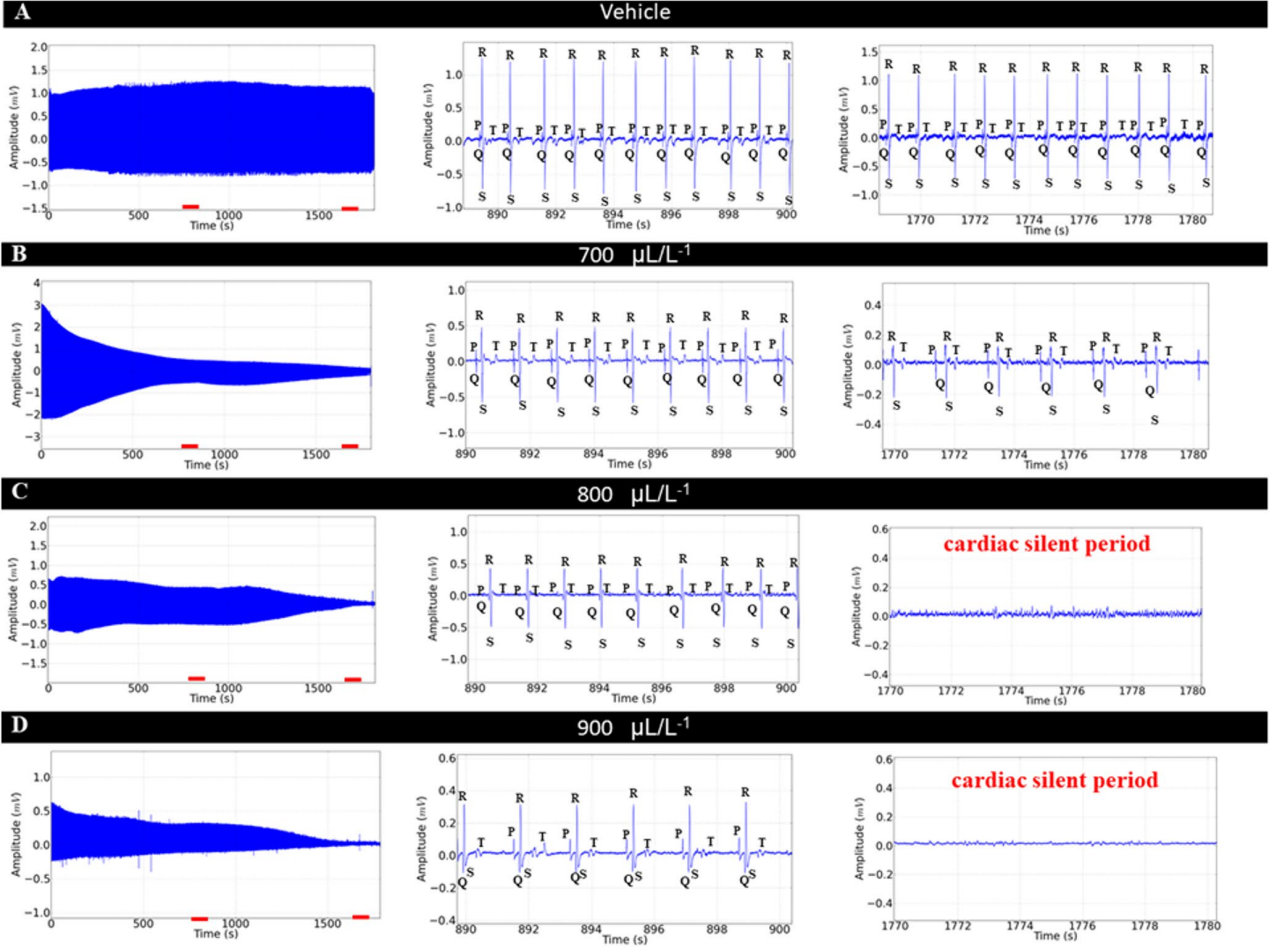
**-** Cardiac activity in tilapia, during immersion of different concentrations of eugenol (left), Amplification of the recording in the last 10 s (890–900 s) (center), demonstration of the amplification of the final 10 s of the recording (1770–1780 s) to identify cardiac deflagrations (right), for the following groups: vehicle group (**A**); Group treated with 700 µL. L^− 1^ (**B**), 800 µL. L^− 1^ (**C**), 900 µL. L^− 1^ (**D**)

During exposure to eugenol at concentrations of 700 µL. L^− 1^, 800 µL. L^− 1^ and 900 µL. L^− 1^ the ECG showed a concentration-dependent decrease in cardiac excitability in the first 15 min of recording when compared to the control and vehicle groups. The group treated with 700 µL. L^− 1^ showed a 31.71% reduction (Fig. 3A and B inthe center). For the group treated with 800 µL. L^− 1^ the percentage reduction in cardiac activity was 44.18% (Fig. 3C, in the center) and for the group treated with 900 µL. L-¹ the reduction in cardiac activity was 58.92% (Fig. 3D, in the center). All experimental groups showed significant reductions in cardiac activity, with statistical differences observed in all cases (***p < 0.001).

In the 30-minute treatment period, the control and vehicle groups were not significantly different (*p* = 0.998). During this period, the group treated with 700 µL. L^− 1^ showed a 53.82% decrease compared to the control, found significant (*p* < 0.001) (Fig. 3B, right). The groups treated with 800 µL. L^− 1^ and 900 µL. L^− 1^ showed no identifiable morphographic elements, indicating a loss of cardiac activity on the ECG (Fig. 3C and D, right).

**Fig. 4:**
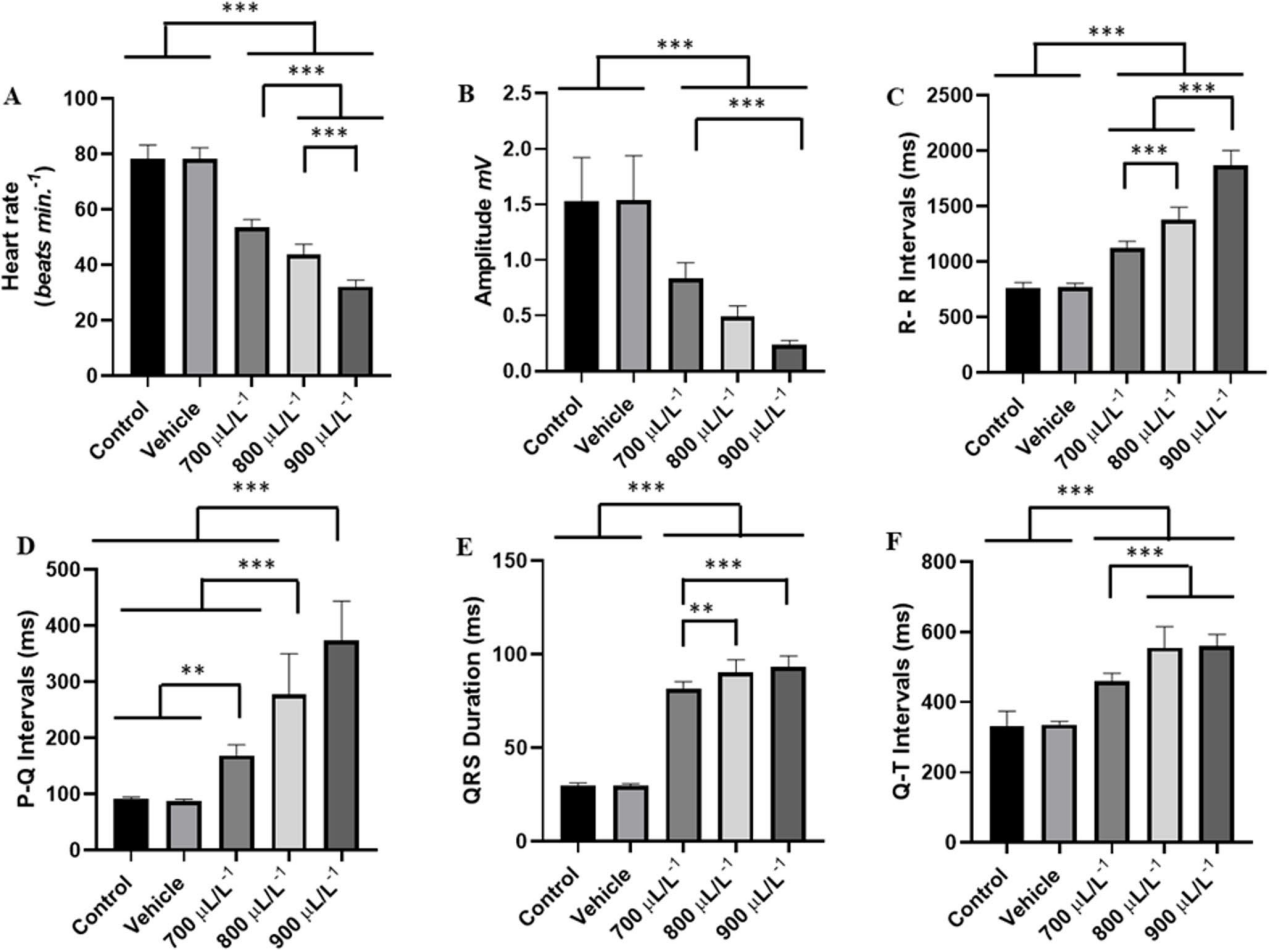
**-** Analysis of the recordings obtained during the first 15 min of eugenol treatment. Mean heart rate values in beats per minute during exposure to eugenol at 700 µL. L^− 1^, 800 µL. L^− 1^, 900 µL. L^− 1^ (**A**), mean values of QRS complex amplitude (mV) (**B**), mean values of R-R intervals (ms) (**C**), P-Q intervals (ms) (**D**), QRS complex duration (ms) (**E**) and QT interval (ms) (**F**). (ANOVA followed by Tukey’s test; *P < 0.05, **p < 0.01, ***p < 0.001; *n* = 9)

Heart rate was significantly affected by the increasing concentrations of eugenol. The group treated with 700 µL. L^− 1^ had a mean of (53.56 ± 2.78 bpm), which was significantly higher than the groups treated with 800 µL. L^− 1^ (43.78 ± 3.66 bpm), and the group treated with 900 µL. L^− 1^ (32.22 ± 2.33 bpm) (*p* < 0.001). The group treated with 900µL. L^− 1^ had significantly lower cardiac activity than the other groups (Fig. 4A).

The mean amplitude of the QRS complex in the control group was 1.526 ± 0.39 mV, with no significant difference to the vehicle group (*p* = 0.999), but it was higher than the other groups. The group treated with 700 µL. L^− 1^ (0.836 ± 0.140 mV) was similar to the group treated with 800 µL. L^− 1^ (*p* = 0.0661). The groups treated with 800 µL. L^− 1^ and 900 µL. L^− 1^ were similar (*p* = 0.265) (Fig. 4B).

The mean RR interval of the control group was 763.3 ± 48.22 ms, similar the vehicle group (*p* = 0.999), but significantly shorter than the other groups. The group treated with 700 µL. L^− 1^ had a mean RR interval of 1122 ± 62.41 ms, less than the groups treated with 800 µL. L^− 1^ (1377 ± 113.7 ms) and the 900 µL. L^− 1^ group (1868 ± 136 ms). The group treated with 900 µL. L^− 1^, was longer than the other groups (Fig. 4C).

The mean PQ interval for the control group was 92.22 ± 263 ms, like the vehicle group (*p* = 0.999) and was shorter than the other groups. The group treated with 700 µL. L^− 1^, was lower than the groups treated with 800 µL. L^− 1^, and 900 µL. L^− 1^. The group treated with 900 µL. L^− 1^ was higher than the other groups (Fig. 4D).

The mean duration of the QRS complex for the control group during induction was 29.67 ± 1.50 ms, like the vehicle group (*p* = 0.999) but shorter than the other groups. For the group treated with 700 µL. L^− 1^ (81.56 ± 3.844 ms) was lower than the groups treated with 800 µL. L^− 1^ and 900 µL. L^− 1^, but the groups treated with 800 µL. L^− 1^ and 900 µL. L^− 1^, showed no statistical difference (*p* = 0.5686) (Fig. 4E).

For the control group, the mean QT interval during the first 15 min of induction was 333.1 ± 41.40 ms, with no difference to the vehicle group (*p* = 0.997) and was shorter than the other groups. The 700 µL. L^− 1^ group (460.2 ± 22.93 ms) was shorter than the other groups. The groups treated with 800 µL. L^− 1^ and 900 µL. L^− 1^ were similar (*p* = 0.998) (Fig. 4F).

**Fig. 5:**
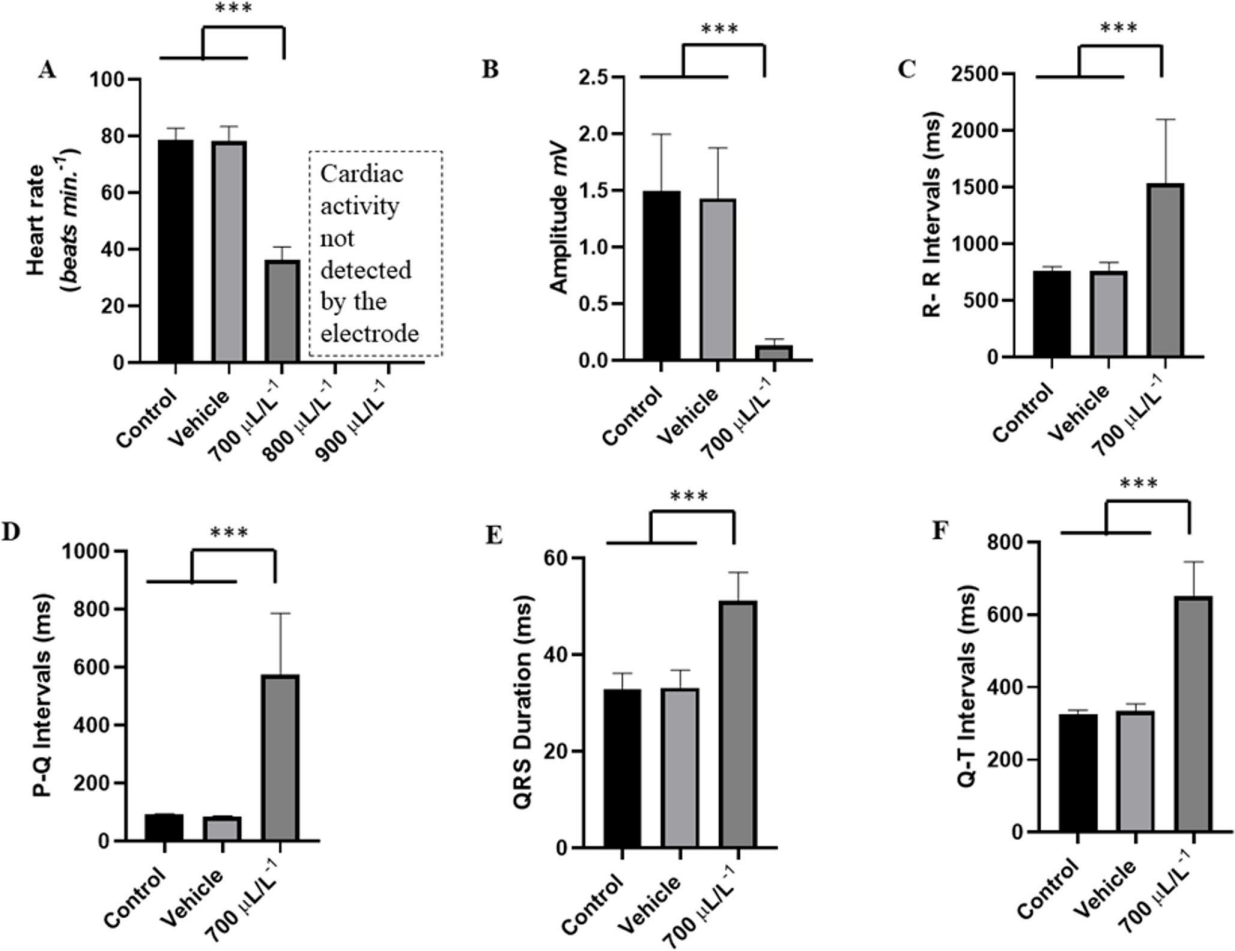
Average values of cardiac parameters during the 30-minute period at high eugenol concentrations: 700 µL. L^− 1^, 800 µL. L^− 1^, 900 µL. L^− 1^. Mean heart rate values (bpm) (**A**); mean QRS complex amplitude values (mV) (**B**); mean R-R values (ms) (**C**); P-Q intervals (ms) (**D**); QRS complex duration (ms) (**E**) and QT interval (ms) (**F**). (ANOVA followed by Tukey’s test; ***p < 0.0001; *n* = 9)

During the final 30-minute contact period (1770–1780 s), cardiac activity was not detected in the groups treated with 800 µL. L^− 1^ and 900 µL. L^− 1^. The Control vs. Vehicle group (*p* = 0.998) was larger than the group treated with 700 µL. L^− 1^ (Fig. 5A). The QRS complex amplitude for the Control vs. Vehicle groups (*p* = 0.929) was higher than the group treated with 700 µL. L^− 1^ (Fig. 5B). The mean RR interval for the control group vs. vehicle group (*p* = 0.999) was lower than the group treated with 700 µL. L^− 1^ (Fig. 5C).

The PQ interval during the 30 min for the control group compared to the vehicle group (*p* = 0.993), however, was shorter than the group treated with 700 µL. L⁻¹ (Fig. 5D). The QRS complex duration in the control group was like that of the vehicle group (*p* = 0.976) but was shorter than in fish treated with eugenol at 700 µL. L⁻¹ (Fig. 5E). The QT interval for the control group was similar to that of the vehicle group (*p* = 0.976), but was shorter than in the group treated with 700 µL. L⁻¹ (Fig. 5F).

**Fig. 6:**
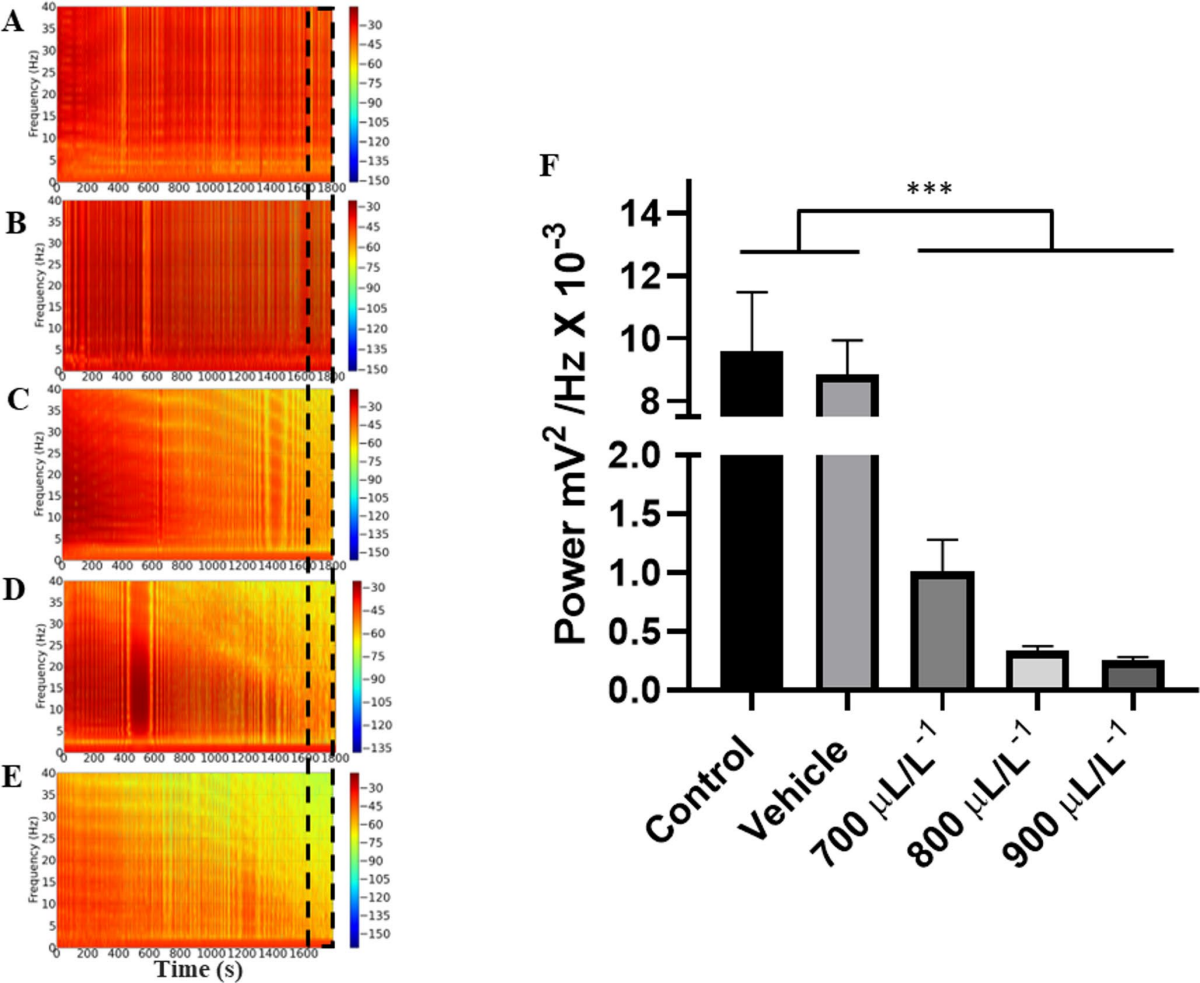
Power distribution values of the groups represented by the energy spectrograms of the electrocardiographic recordings during the final 30 s (1770–1800 s) of the 30 min of recording of cardiac parameters in the following groups: Control (**A**); Vehicle (**B**); 700 µL. L^-1^ (**C**), 800 µL. L^-1^ (**D**) and 900 µL. L^-1^ (**E**). Mean power values of cardiac activity (**F**). (ANOVA followed by Tukey’s test; ***p < 0.0001; *n* = 9

The power distribution spectrograms show a decrease in the power of cardiac activity for the higher concentrations of eugenol (Figs. 6A, B, C, D and E), which can be better observed in the linear power graph, where the control group showed an average linear power like the vehicle group (*p* = 0.498). However, these groups were larger than the other groups (*p* < 0.001). The groups treated with 700 µL. L^− 1^, 800 µL. L^− 1^ and 900 µL. L^− 1^ were similar (*p* = 0.4811).

## Discussion

Several anesthetics have been studied and recommended in the literature for the practice of euthanasia in fish (Skar et al. 2017; Davis et al. 2022). Eugenol is widely known for its effectiveness as an anesthetic in fish management (Schroder et al. 2022; Ventura et al. 2020; Fernandes et al. 2017). The use of eugenol for euthanasia is described in the literature (Ayala-Soldado et al. 2023; Neiffer and Stamper 2009), but there are no records of its application in Nile tilapia. This study investigated the evolution of the ECG with the use of high concentrations of eugenol for the euthanasia of Nile tilapia.

Eugenol has been shown to cause hemodynamic changes when used in doses higher than the safe concentration window in Nile tilapia (da Paz et al. 2024). This finding can be explained by eugenol’s interactions with the neurotransmitter GABA (Lee et al. 2015), which increases parasympathetic activity. As a result, there is a release of acetylcholine in the heart by vagal neurons and consequent activation of M2 muscarinic receptors, which causes hyperpolarization of the sinoatrial node cells and causes bradycardia (Dolejsí, Janoušková & Jakubík et al., 2024).

The concentrations required for anesthesia vary depending on the species and other factors, but can be as low as 17 mg/L for some species such as the Danio rerio. However, concentrations 10 times the upper limit of the anesthetic concentration correspond to the doses required for euthanasia (Neiffer and Stamper 2009). Fish should remain in the anesthetic solution for at least 10 min after the cessation of opercular movement (Gladden et al. 2010; National Toxicology Program 2010). However, our study demonstrated that a concentration 10 times the anesthetic dose can cause euthanasia. However, the contact time must be greater than 10 min in an immersion bath. This is because, according to observational data on the cessation of opercular movement, electrophysiological data showed that the heart continues to function for a long time. Thus, respiratory arrest may erroneously lead the observer to believe that it is the end point.

This mechanism agrees with the electrocardiographic findings in the results. The 15 min immersion bath in the animals showed a progressive decrease depending on the high concentration of eugenol when compared to the control and vehicle groups; and during the 30 min period, the concentration of 700 µL. L^-1^ showed a 53.82% reduction in frequency compared to the control group. The groups treated with 800 µL. L^-1^ and 900 µL. L^-1^ showed cardiac silence within 30 min of contact, as recorded on the ECG. It is worth noting that heart rate variability is described as a sensitive and non-invasive parameter for obtaining sympathovagal stimuli during euthanasia (Gehlen et al. 2020). These observations suggest that the practice of euthanasia with eugenol in high concentration (from 800 µL. L^-^¹) is only effective when used within 30 min.

Electrocardiography showed a dose-dependent increase in the PQ interval, which coincides with a decrease in heart rate over a 15-minute period. The P wave represents the sinus rhythm, responsible for generating the cardiac impulse (Chandler et al. 2009; Bagliani et al. 2003). It has been established that heart rate is dependent on the PQ interval (Malik et al. 2008). A greater increase in the PQ interval was observed at the concentration of 700 µL. L^− 1^ at the 30-minute recording interval compared to the 15-minute interval and a loss of the PQ interval at the concentrations of 800 µL. L^− 1^ and 900 µL. L^− 1^ at 30 min of recording, suggesting that the administration of eugenol causes a change in the electrical conduction of the heart, reflected in the prolongation of the PQ interval. It is worth noting that other wave elements were not recorded at concentrations of 800 µL. L^− 1^ and 900 µL. L^− 1^ within 30 min.

It is common for fish to show a prolongation of these intervals when subjected to anaesthetics due to a reduction in physiological mechanisms such as breathing and heartbeat, because most anesthetics used in fish are GABA modulators, allowing desensitization to pain and external stimuli (da Paz et al. 2024; Reis et al. 2024; Vilhena et al. 2022; Vieira et al. 2023), so when the aim is to achieve euthanasia, this prolongation is expected to be much longer due to the bradycardia caused by the long contact time with the anesthetic agent (da Paz et al. 2024).

An important fact to add is that eugenol can form a coating on the gill epithelium that potentially blocks the diffusion of gases (Sladky et al. 2001). It has been documented that exposure can cause necrosis in gill tissue (Ayala-Soldado et al. 2023; Afifi et al. 2001). Other possible reasons for ventilatory failure and spinal cord collapse in fish can be caused by neurotoxic or hepatotoxic effects (Sladky et al. 2001).

The safe anesthetic concentration window for eugenol in Nile tilapia is between 50 µL. L ^-1^ and 100 µL. L ^-1^, and at concentrations between 100 µL. L^-1^ and 150 µL. L^-1^ it is possible to induce states of deep anesthesia (da Paz et al. 2024). According to Ross and Ross (2008), for euthanasia practices to be effective, the anesthetic concentration must be increased by five to ten times the amount used in each species. The results obtained showed that immersing Nile tilapia in a concentration of 800 µL. L^-1^ of eugenol for 20 min can cause asystole. Cardiac asystole occurs after brain death in fish, since fish myocardial cells use local glycogen reserves as an energy source and do not require blood glucose (Stetter 2001).

Compared to other anesthetics considered potentially effective for euthanasia in fish, such as tricaine, it is recommended that euthanasia be performed by immersion for 5 to 10 min after the cessation of opercular movements, with doses ranging from 250 to 500 mg. L^− 1^. However, this time and dosage are questionable, and even when higher doses are used for longer periods, it does not always guarantee that the fish died, requiring the monitoring of other parameters to confirm death, such as electrocardiographic assessment (Balko et al. 2018; Neiffer and Stamper 2009; Sherrill et al. 2009). Studies involving zebrafish larvae (*Danio rerio*) subjected to high concentrations of MS-222 (300 and 600 mg. L^− 1^) have already reported the ineffectiveness of this product in inducing euthanasia, with recovery observed in the animals even after 1 h of exposure (Strykowski and Schech 2015) Eugenol proved to be more effective than MS-222 regarding the time taken to induce sacrifice in zebrafish (Ayala-Soldado et al. 2023).

However, this study has limitations related to the size of the animals used and the lack of long-term recovery assessment to determine if cardiac activity recovers when placed in water without anesthetic for recovery. Additional environmental or metabolic stressors that may influence the response to anesthetic should also be taken into consideration.

## Conclusion

Thus, with the results obtained in this study on the use of eugenol, we have obtained a standard anesthetic agent for euthanasia in *O. niloticus* with its respective dose and effective time of sacrifice for laboratory activities. In the final 30 s of recording (1770–1800 s), no cardiac activity was detected for 800 µL. L^-1^ or 900 µL. L^-^¹. This demonstrates a similar endpoint of cardiac arrest for both concentrations. However, the study highlights a dose-dependent progressive decrease in heart rate during the early exposure periods. The group treated with 800 µL. L^-1^ showed a 44.18% reduction in cardiac activity at 15 min, while the group treated with 900 µL. L^-1^ showed a greater reduction of 58.92% at the same time point. Although the result is the same, the higher concentration of 900 µL. L^-1^ may result in a more rapid reduction of cardiac activity during the initial minutes of exposure, potentially shortening the time until loss of consciousness and nociceptive perception. Therefore, we highlight the dose of 800 µL. L^-1^ as effective and safe for euthanasia of tilapia for the 30-minute exposure time However, this fact may cause possible discomfort to the animal until death. Other natural anesthetic agents for the purpose of euthanasia should be investigated to enlighten the scientific community about the sacrifice of fish in a laboratory setting.

## Data Availability

No datasets were generated or analysed during the current study.
